# Investigating magnetic and optical properties and morphology of a nanocomposite of intercalated layered material and polymer compounds

**DOI:** 10.1038/s41598-026-52585-6

**Published:** 2026-05-19

**Authors:** Amin A. El-Meligi, Eman. H. Ahmed, Amal M. Abdel-karim, Ashraf A. Abbas, Bassem S. Nabawy

**Affiliations:** 1 Physical Chemistry Department, National Research Centre, 33 El Bohouth St., Dokki, P.O.12622, Giza, Egypt; 2 Polymers and Pigments Department, Chemical Industries Institute, Advanced Materials and Nanotechnology Group, National Research Centre, 33 El Bohouth St., Dokki, P.O.12622, Giza, Egypt; 3 Chemistry Department, Faculty of Science, Cairo University, P.O.12613, Giza, Egypt; 4 Geophysical Sciences Department, National Research Centre, 33 El Bohouth St., Dokki, P.O.12622, Giza, Egypt

**Keywords:** Layered material, Nanocomposite, Doping polymers, XRD, Magnetic properties, Chemistry, Materials science, Nanoscience and technology

## Abstract

The study focuses on doping polyvinyl pyrrolidone (PVP), polyacrylic (PAC), and nanosilica (SiO_2_) into the layered material FePS_3_ intercalated with 1,3-bis(amino-5-phenyl-1,2,4-triazol-3-ylsulfanyl) propane (FePS_3_-BAPT). Different ratios of FePS_3_-BAPT were incorporated into the polymers (PVP, PAC, and SiO_2_) to form a nanocomposite. This nanocomposite was characterized by a number of tools: X-ray diffraction (XRD), Fourier-transform infrared spectroscopy (FTIR), Scanning Electron Microscopy supported by Energy Dispersive X-ray analysis (SEM-EDX), and Transmission Electron Microscope (TEM). Magnetic and optical properties of the prepared nanocomposite were evaluated. XRD analysis indicates that the ratio of doped materials has affected the detection of FePS_3_-BAPT. The XRD patterns also reveal the amorphous nature of the polymers. The nanocomposite crystallinity is about 1.5%, while the amorphous content percentage is about 98.5%. TEM results confirm the amorphous structure of the polymers within the nanocomposite. The magnetic properties of FePS_3_-BAPT have been affected after doping with the PVP/PAC/SiO_2_; this shift is due to the lack of charged ions, the cluster size, and the amorphous nature of the polymers. According to the magnetic hysteresis loop, the magnetic behavior shifted from paramagnetic to diamagnetic. This shift is because of the revealing of the amorphous nature of polymers and the absence of unpaired electrons. The nanocomposites of FePS_3_-BAPT/PVP/PAC/SiO_2_ (H11, H13, and H19) show an increase in band gap energy; this increase is due to strong interfacial interactions between the layered FePS₃ structure and polymer matrix and also the effect of insulation in the presence of SiO₂ nanoparticles.

## Introduction

Researchers are interested in mixing iron phosphorus trisulfide (FePS_3_) with organic compounds because the new materials created have many benefits. When organic compounds are added between the layers of FePS_3_, it increases the space between the layers, which significantly alters the material’s magnetic, optical, and electrical properties^1–4^. The added compounds can be mixed with polymer materials, which may create new nanocomposites that significantly change their magnetic, electrical, and optical properties^5,6^. The good electrical and magnetic behaviors of the conducting polymers can be a robust factor to produce new composites when combined with the layered chalcogenide nanomaterials, such as FePS_3_, MnPS_3_, NiPS_3_, etc. As stated, polymer nanocomposites (PNC) have the same properties as the polymers, such as low cost, flexibility, and light weight. Furthermore, the quantum-size impact and tiny-size implementations contribute to the retention of useful features in PNC. Additionally, the surface/boundary effect of PNC shows adjustable electrical, optical, mechanical, and biological properties, which can be tailored for specific applications in fields such as optoelectronics and biomedicine^7^. Polymers and polymer nanocomposites present a notable candidate of adaptable properties that lead to a vast array of potential applications, such as optoelectronics. As reported, polyvinyl alcohol and its nanocomposite films could be applied for optoelectronic applications because of their optical and electronic properties^8–10^. Furthermore, nanocomposite films of polyvinyl butyral doped with other materials such as metal nanoparticles or conductive polymers, were prepared for potential optoelectronic applications due to their enhanced optical and electrical properties.

Some studies reported the formation of organic-inorganic composites. For example, poly(methyl methacrylate) and metal oxides (e.g., titanium oxide TiO_2_, silicon oxide SiO_2_, iron oxides, zinc oxide ZnO, gallium oxide Ga_2_O_3_, nickel oxide NiO, copper oxide CuO, and cerium oxides) can create organic-inorganic nanocomposites. This is because of their remarkable magnetic, optical, electrical, catalytic, and mechanical properties^11–13^. A nanocomposite made of polypyrrole doped with SiO_2_, and FePS_3_ was processed, and there was a change in the conductivity behavior of FePS_3_^14^. Measuring the magnetic properties of polymer compounds doped with intercalated layered materials (FePS_3_-BAPT) provides a valuable trend for multidisciplinary research integrating the layered nanomaterials (MPX_3_), organic chemistry, and polymer chemistry. This field of materials science, intercalation chemistry, and doping chemistry can pave the way for synthesizing novel nanocomposites with tailored characteristics for advanced applications. Furthermore, the reformed magnetism properties of hybrid nanomaterial-polymer-organic systems can advance their applications and create opportunities for technological innovation.

In fact, FePS_3_ is a well-defined layered magnetic semiconductor material, but the strategies to simultaneously tune its magnetic and optical properties via organic compound intercalation and doping of polymer/nanosilica (SiO_2_) need more exploration^15,16^. Accordingly, this study presents novel doping of FePS_3_-BAPT with polymers (PVP and PAC) and SiO_2_ in carefully selected ratios to systematically control interlayer expansion, interfacial electronic interactions, and polymer coverage, optimizing both magnetic modification and widening of the band gap^17,18^. Based on magnetic and optical behavior and modification, the novelty of this study is to address a key gap in the design of multifunctional nanocomposites.

This study investigates the results of doping PVP and PAC with two nano-inorganic materials, SiO_2_ and FeSP_3_-BAPT, to produce organic-inorganic nanocomposites and measures the magnetic properties of the resulting nanocomposite.

## Experimental techniques

### Preparation of FePS_3_-BAPT

The FePS_3_ layered nanomaterial was prepared using stoichiometric amounts of high-purity elements (99.99%), Fe, P, and S. Intercalation of the FePS_3_ with BAPT was processed and was characterized elsewhere^5^.

### Chemical modification of polyvinylpyrrolidone (PVP) and polyacrylic (PAC)

**Scheme 1:**
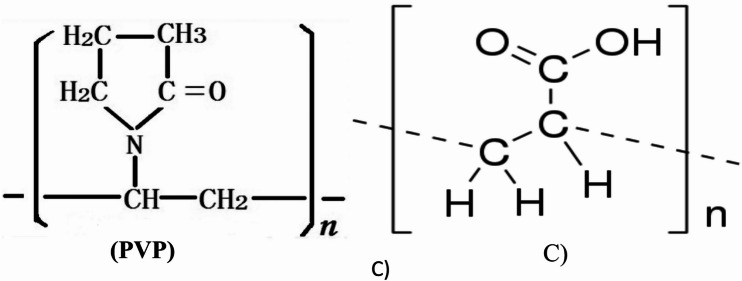
Chemical structure of PVP and PAC.

The chemical structure of PVP and PAC are shown in scheme 1. Different ratios of the polymers PVP and PAC as well as SiO_2_ were doped into the intercalated compound, FePS_3_-BAPT. The first ratio is 1 FePS_3_-BAPT: 3 PVP: 3 PAC: 13 SiO_2_ for the H11 sample, the second ratio is 1 FePS_3_-BAPT: 5 PVP: 5 PAC: 11 SiO_2_ for the H13 sample and the third ratio is 1 FePS_3_-BAPT: 7.5 PVP: 7.5 PAC: 12 SiO_2_ for the H19 sample.

### Characterization, magnetic, and optical testing

X-ray diffraction (XRD) patterns were recorded by the X-ray diffractometer using monochromatized CuK radiation of wavelength = 1.54056 A^o^ at 40 kV and 30 mA. A Python program applied quantum calculation to determine the percentage of crystallinity and percentage of amorphousness. The FTIR transmittance spectra were generated on a JASCO FT-IR 6100, which was manufactured in Japan utilizing KBr pellets. SEM in conjunction with Energy Dispersive X-ray analysis (EDX) was applied to test the morphology and elemental analysis of the materials. SEM-EDX was performed with an FEI Quanta 200 FEG-SEM equipped with a Bruker Si (Li) EDX detector. The images from transmission electron microscopy (TEM) and high-resolution transmission electron microscopy (HTEM) were performed using a Jeol (JEM-2100 PLUS) at an accelerating voltage of 200 kV. A Lakeshore 7407 vibrating sample magnetometer (VSM) was used to measure the magnetic properties. The optical behavior was tested using an absorption spectrum as a function of wavelength under ambient settings using a V-570 optical spectrophotometer (Jasco, Japan) with a spectral range of 250–600 nm.

## Results and discussion

### XRD measurements

The XRD patterns of the layered material FePS_3_ and the intercalated compound FePS_3_-BAPT were previously measured and discussed^5,19^. In this study, the novel nanocomposite (FePS_3_-BAPT)-PVP/PAC/SiO_2_ was characterized using XRD.

**Fig. 1:**
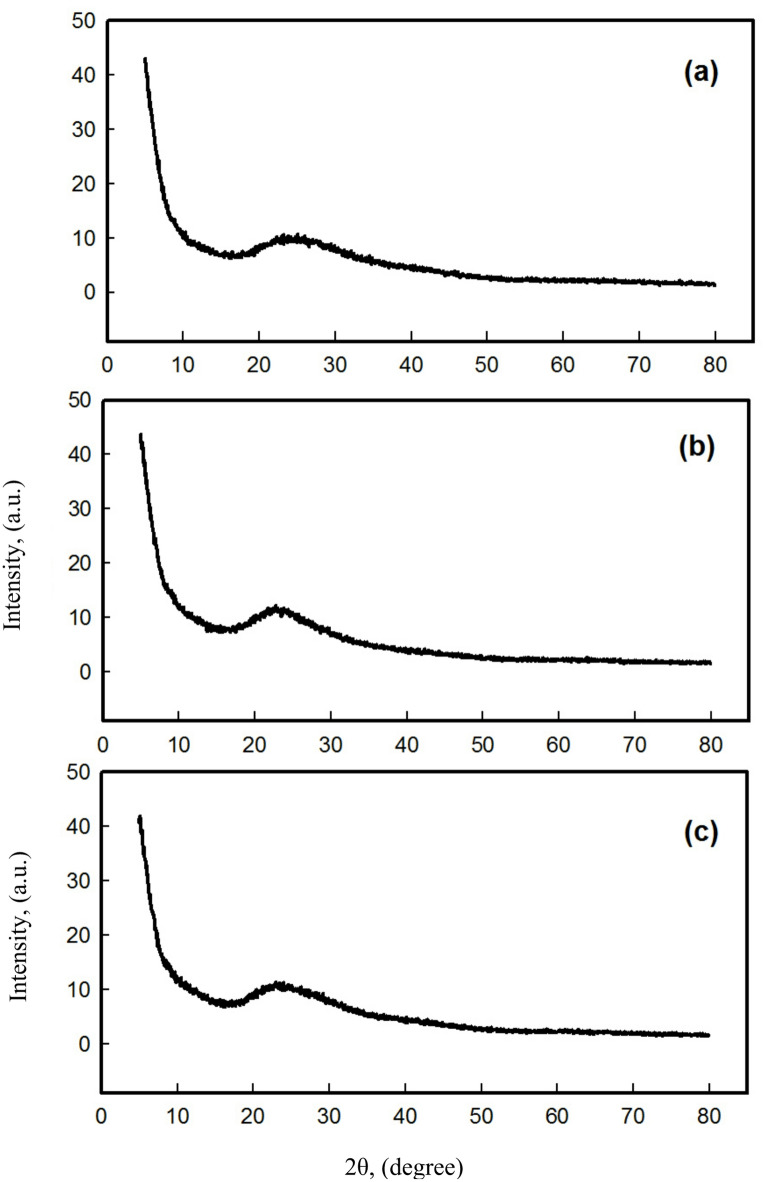
(**a**) H11 composite, (**b**) H13 composite, and (**c**) H19 composite.

Figure 1 shows the XRD patterns of different mixtures of the (FePS_3_-BAPT)-PVPP/PAC/SiO_2_ nanocomposite, labeled (H11, H13, and H19). The XRD patterns show the amorphous nature of the nanocomposite polymer, revealing the crystalline nature of the FePS_3_-BAPT. Accordingly, the well-defined hkl phases of FePS_3_-BAPT did not appear in the XRD pattern. A clear broad peak appears in the 2θ range of 20–30°, which indicates that there is an amorphous structure of SiO_2_, PVP, and PAC components^20–22^. Accordingly, the matrix of the nanocomposite, FePS_3_-BAPT/PVP/PAC/SiO_2_, has been created through the physical interaction between the components. The degree of crystallinity was calculated using a Python program. The actual XRD data (2θ and intensity) were used in the calculation. Based on the actual XRD data, the crystallinity percent (Cr%) of the material was calculated using Eq. 1, and the degree of amorphousness was calculated using Eq. 2^23,24^.

1 $${\text{Cr }}\% {\text{ }}={\text{ 1}}00{\text{ }} \times {\text{ Ac}}/({\mathrm{Ac}}\,+\,{\mathrm{Aa}})$$

Where, Ac is the area under crystalline peaks, and Aa is the area under the amorphous region.

2 $${\text{Amorphousness }}\% {\text{ }}={\text{ 1}}00{\text{ }} - {\text{ Crystallinity }}\%$$

In all nanocomposite samples (H11, H13, and H19), the crystallinity percentage is about 1.5%, and the percentage of amorphousness is about 98.5%. This indicates that the material is predominantly amorphous because of the presence of doped polymer compounds, with only a small crystalline content; this is due to the presence of a small amount of the intercalated compound FePS_3_-BAPT. In fact, transforming from crystalline to amorphous form is usual and occurs under certain conditions; for example, silicon phthalocyanine dichloride (SiPcCl_2_) was polycrystalline and transformed to an amorphous form for pristine and annealed thin films^24,25^.

### SEM measurements

**Fig. 2:**
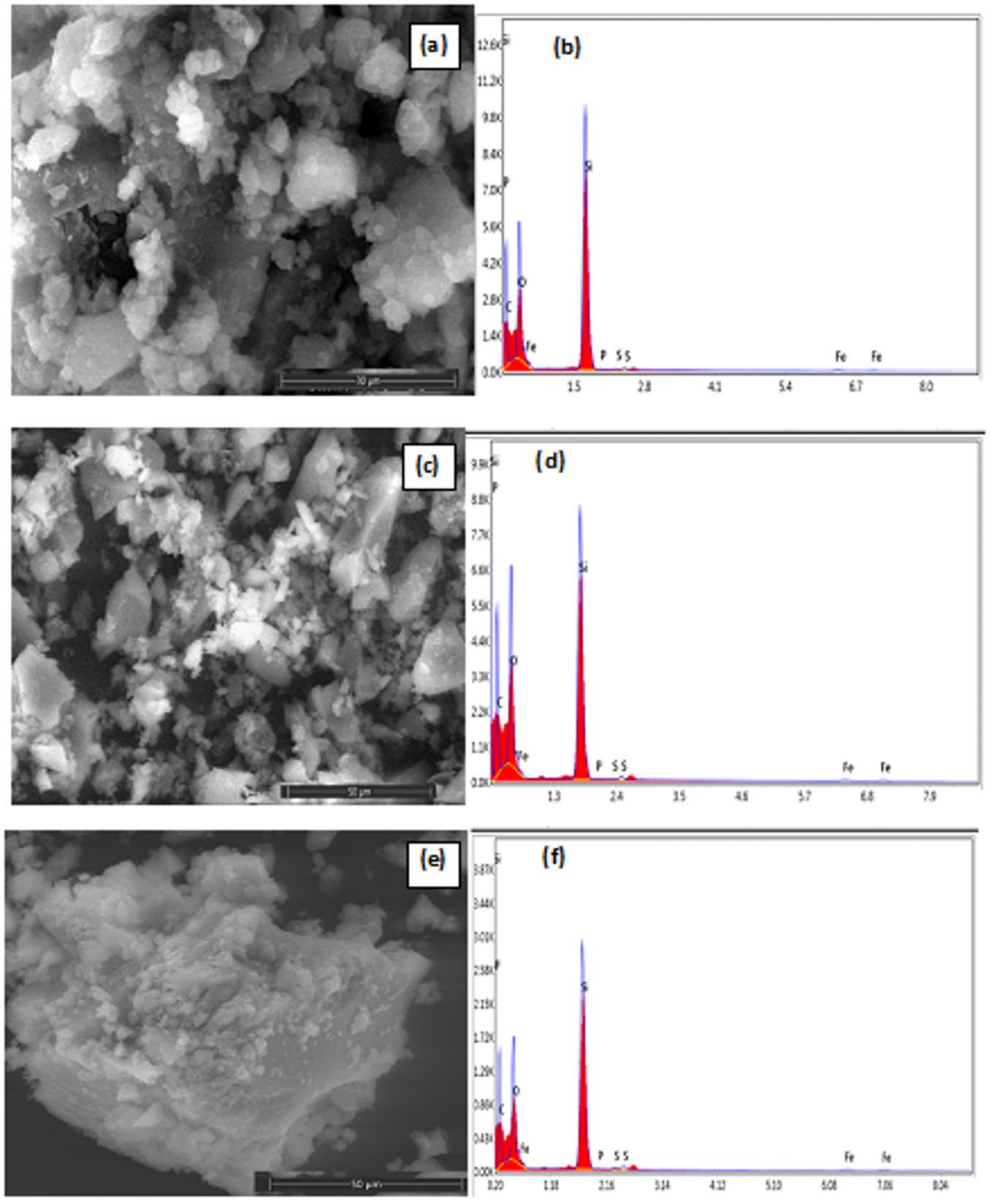
SEM of the nanocomposites FePS_3_-BAPT/PVP/PAC/SiO_2_: (**a**) H11 (**c**) H13, (**e**) H19 and their corresponding EDX spectra, (**b**) H11, (**d**) H13, (**f**) H19.

The SEM images display a close-up view of the surface morphology, revealing a complicated structure made up of aggregated particles and polymer molecules agglomerated on the FePS_3_-BAPT surface, as shown in Fig. 2 (a, c, e). The aggregation and agglomeration of polymer molecules suggest amorphous growth on the FePS_3_-BAPT surface. This behavior applies to the three ratios of polymers. Additionally, the irregular agglomeration of doped polymer molecules (PVP-PAC) indicates enhanced attachment of nanoparticles to FePS_3_ and SiO_2_. It is reported that the distribution of the granules is constant throughout the nanocomposite material and polypyrrole^26^. This behavior can be applied in this research, where the granules of PVP-PAC distribute constantly throughout the matrix of nanomaterials of FePS_3_-BAPT and SiO_2_. This way of distribution helps the nanomaterials of FePS_3_-BAPT to maintain their magnetic behavior. The intercalated compound of FePS_3_-BAPT was tested using energy-dispersive X-ray spectroscopy (EDX). The elemental identification indicates the presence of Fe, P, C, N, and S in the prepared material^5^, as shown in Table 1. The EDX results show the presence of the elements of the nanocomposites of all ratios, as shown in Fig. 2 (b, d, and f). The nanocomposite contains the elements Fe, P, S, Si, C, O, and N. The existence of PVP-PAC is indicated by the presence of the elements C, O, and N (Fig. 2b, d, and f).

**Table 1:** Elements weight% and atomic % of EDX of H11, H13, and H19.

Element	H11		H13		H19	
	Weight%	Atomic %	Weight%	Atomic %	Weight%	Atomic %
C K	42.11	53.63	41.50	52.18	47.06	56.69
O K	36.32	34.73	40.48	38.21	41.35	37.40
Si K	21.07	11.47	17.66	11.50	11.26	5.80
P K	0.07	0.03	0.05	0.02	0.04	0.02
S K	0.09	0.04	0.095	0.02	0.12	0.05
Fe K	0.35	0.10	0.26	0.07	0.17	0.04

### TEM measurements

**Fig. 3:**
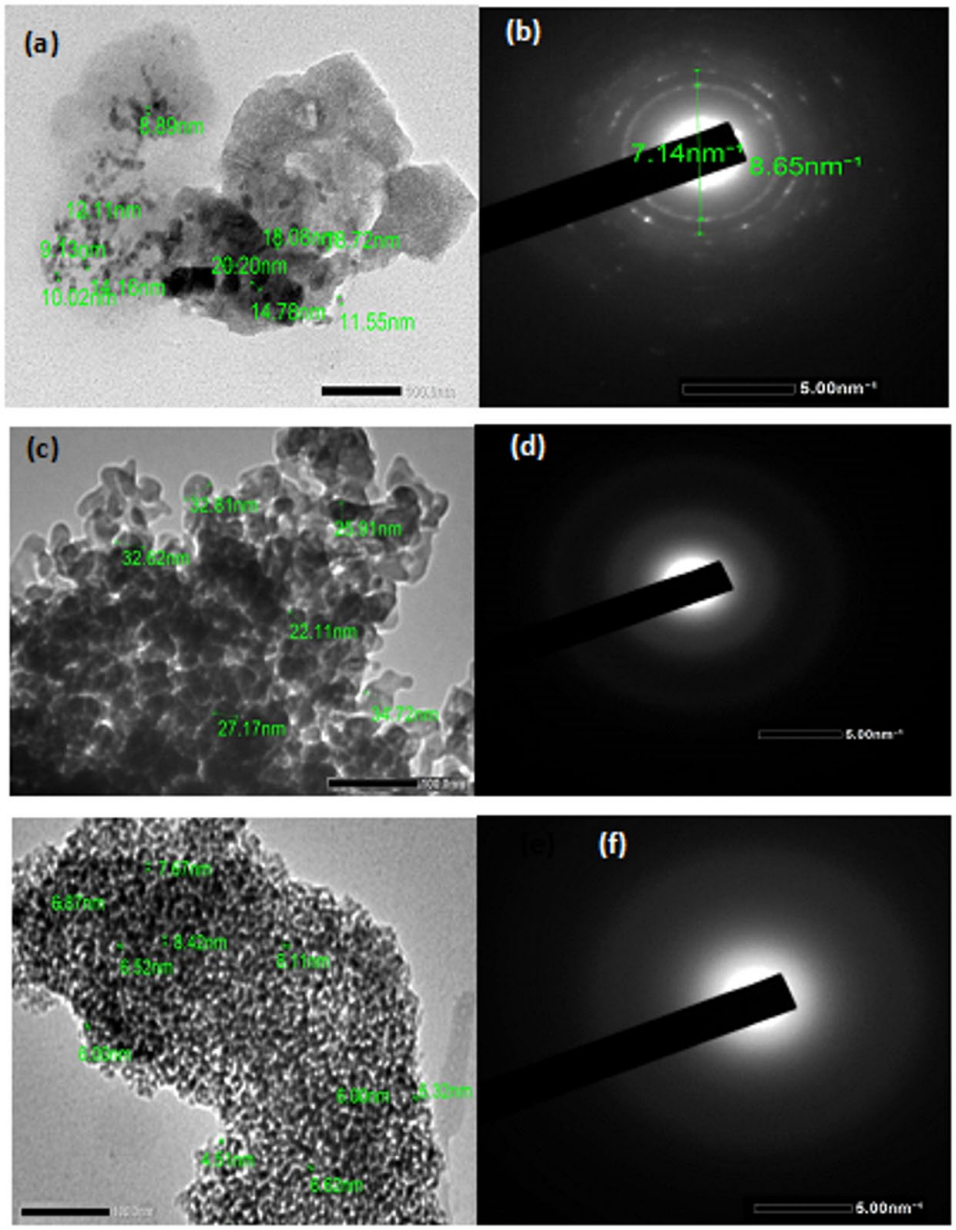
TEM images of the nanocomposite samples (**a**) H11, (**c**) H13, and (**e**) H19, and their corresponding selected area electron diffraction (SAED).

TEM testing was used to evaluate the cluster size and the crystalline structure morphology of the prepared nanocomposite FePS_3_-BAPT/PVP/PAC/SiO_2_, as shown in Fig. 3(a-d).

In the case of H11, the TEM analysis shows that the nanocomposite exhibits agglomeration with a spherical shape and an average cluster size in the nanoscale range of 8.72 nm to 20.20 nm, as shown in Fig. 3a. As in Fig. 3b, the SAED pattern reveals the amorphous nature of the material.

In the case of H13, the TEM testing indicates that the nanocomposite exhibits aggregation with a spherical shape and an average cluster size in the nanoscale range of about 22 nm to 33 nm, as shown in Fig. 3c. Additionally, Fig. 3d shows the SAED pattern, which reveals the amorphous nature of the material.

In the case of H19, the TEM image shows the spherical shape of the nanocomposite clusters with smaller dimensions, ranging from 4.51 to 8.52 nm, as shown in Fig. 3e. Furthermore, Fig. 3f shows the SAED pattern of the nanocomposite, which reflects the amorphous feature of the material.

As it is known, amorphous materials are characterized by a random atomic arrangement. The SAED patterns of the nanocomposites (H11, H13, and H19) do not show well-defined crystal planes; instead, they show broad and faint rings, which reflect the disordered atomic structure typical of amorphous phases^27,28^.

### IR measurements

**Fig. 4:**
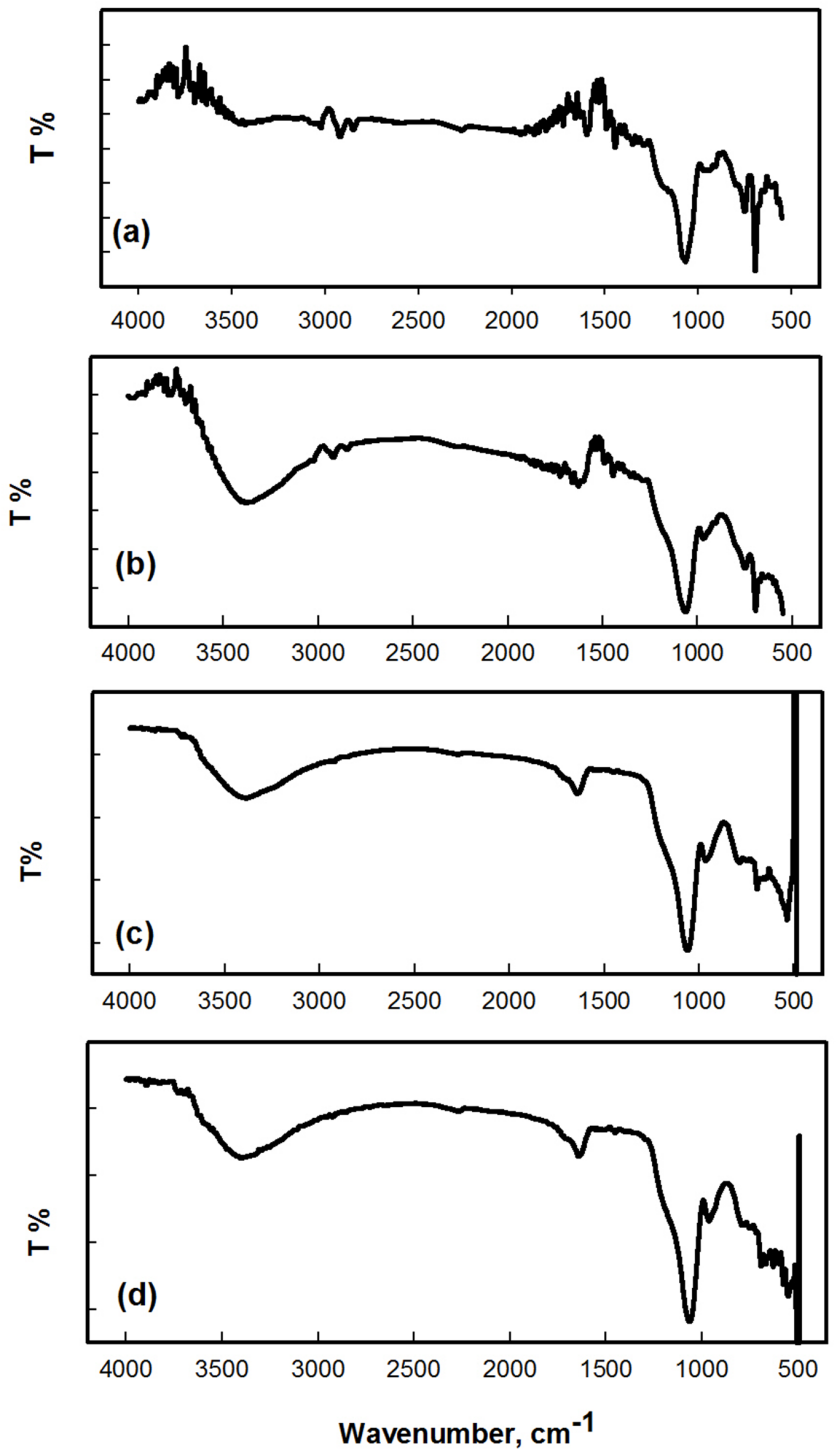
IR spectra of the SiO_2_ (**a**) and the FePS_3_-BAPT/PVP/PAC/SiO_2_ nanocomposites with its different ratios: (**b**) H11, (**c**) H13, and d) H19.

The FTIR spectra of the nanocomposite show notable shifts in characteristic functional group vibrations, indicating interfacial interactions among FePS_3_-BAPT, PVP, PAC, and SiO_2_. The IR spectra of SiO_2_, and the nanocomposites (H11, H13, and H19) are shown in Fig. 4 (a-d). The IR asymmetric stretching band of FePS_3_-BAPT corresponding to the (PS_3_)^2−^ group appears in the spectrum in the range 550–620 cm^−1^^29^. The broad stretching band in the range of 3700 and 3200 cm^−1^, attributed to hydroxyl (-OH) and amino (-NH) groups, is observed, indicating the presence of water and polymer compounds in the nanocomposites, as shown in Fig. 4 (b, c, d)^30,31^.

There is a small absorption peak of the PVP/PAC at about 1650 cm^[–1^; this peak appears in the three samples (H11, H13, and H19). The bands at 1050 cm^−1^ associated with the C-H/N-H in-plane band, the band at 1217 cm^−1^ associated with C-N stretching vibrations, the bands at 1566 cm^−1^ associated with C-H in-plane stretching and ring stretching modes, respectively, and the band at 1637 cm^−1^ associated with C-N stretching all support the structure of PVP/PAC, as previously reported^32^.

The IR spectrum of SiO_2_ (Fig. 4a) shows an asymmetric stretching vibration for the Si-O-Si group with a slight shift at 1105 cm^−1^; as reported, the Si–O–Si asymmetric stretching band of the SiO₂ nanoparticles is nearly detected at about 1080–1100 cm^−1^; therefore, the slight shift and broadening of the band in the composite supports the formation of hydrogen bonding interactions between polymer chains and silica surfaces^33,34^. A stretching band for Si-OH is noticed at 951 cm^−1^, and two stretching bands for Si-O is noticed at 802 cm^−1^ and 468 cm^−1^^35^. The stretch band corresponding to the C = O group of both PVP and PAC appears at approximately 1658 cm^−1^. There is a slight shift in the band of C = O due to interaction among components of the nanocomposite. It was reported that in polymers PVP and PAC, the carbonyl group (C = O) stretching bands typically appear at about 1655–1660 cm^−1^ for the PVP and 1705–1715 cm^−1^ for the PAC, respectively. However, in the nanocomposite, these bands shift to slightly lower wavenumbers, suggesting coordination of carbonyl oxygen atoms with Fe centers and hydrogen bonding with surface silanol (Si–OH) groups of SiO₂. As reported, FTIR band shifts associated with metal-polymer and oxide-polymer interfacial interactions have been stated in metal-polymer and silica-polymer nanocomposites, where such interactions were correlated with improved electronic coupling and modified optical properties^36,37^. These spectral changes provide direct evidence of interfacial bonding, supporting the mechanism proposed in Sect. 3.6 for the observed widening of the optical band gap.

### Magnetic measurements

**Fig. 5:**
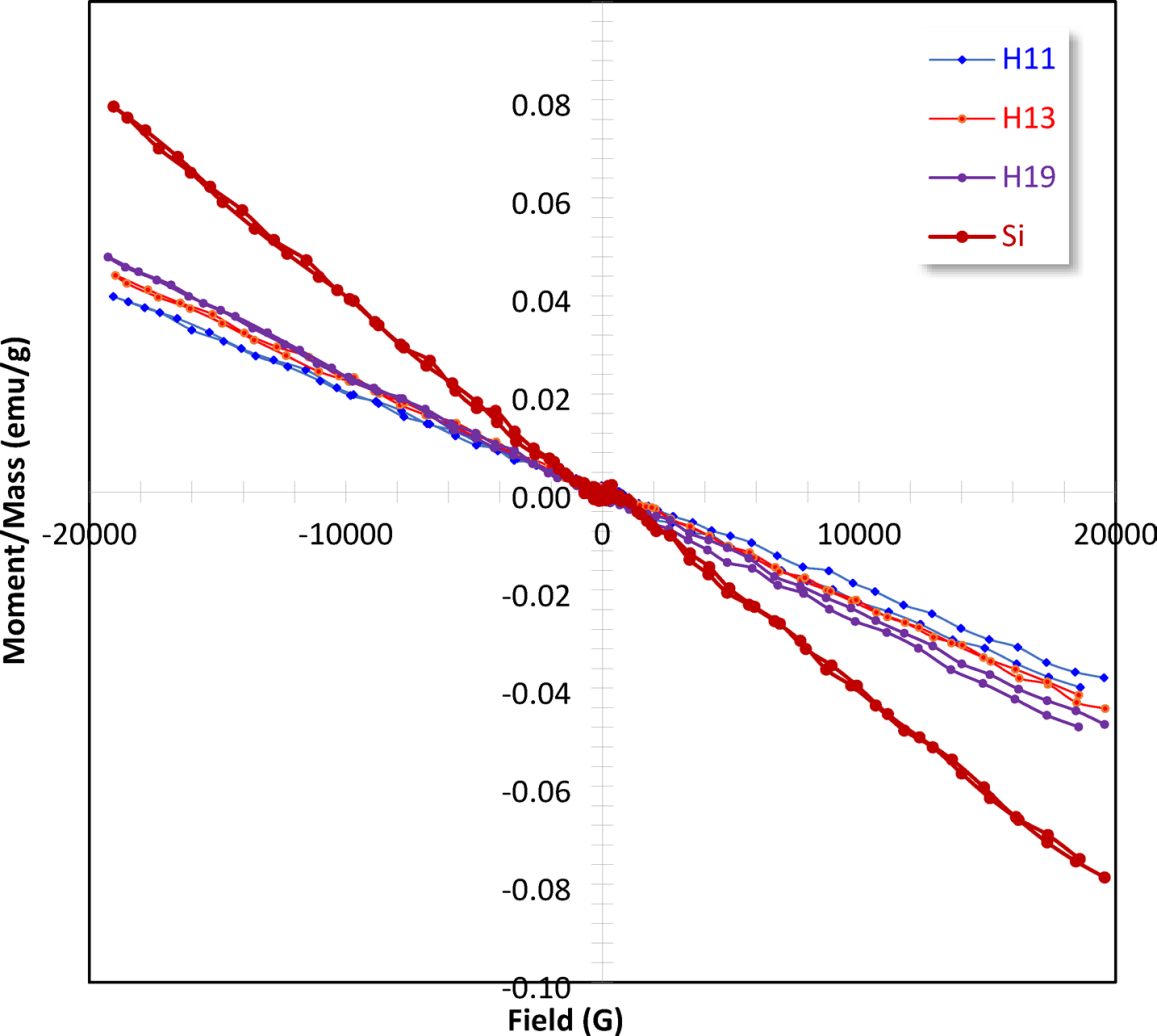
Magnetic hysteresis loops of the studied samples.

The magnetic properties of FePS_3_-BAPT were measured, and it exhibited paramagnetic behavior^5^. The results of the composite of FePS_3_-BAPT doped with different ratios of PVP-PAC are denoted as H11, H13, and H19. There is a linear shape of the magnetic behavior (M-H relation) of the composites, which is similar to the magnetic behavior of FePS_3_-BAPT, but the curve presents a linear decrease (negative side), indicating a weak repulsion from the applied magnetic field, as shown in Fig. 5. Accordingly, the paramagnetic behavior of the nanocomposite FePS_3_-BAPT has changed after doping with the polymers to diamagnetic^38,39^.

As explained, paramagnetic materials have atoms with unpaired electron spins, oriented randomly in the absence of external magnetic fields^38,39^. In fact, the magnetic hysteresis loop can be applied successfully to differentiate between various types of magnetic behavior, i.e., to differentiate between the diamagnetic, paramagnetic, super-, ferri-, and ferromagnetic, as stated by many authors^40–43^. Generally, some factors, such as unpaired electrons, magnetic ion spacing, carrier density, morphology, size, structure, and surface disorder, affect the magnetic behavior^44–46^. The doped polymers don’t have unpaired electrons or a magnetic ion, which means that doping the PVP-PAC polymers with the FePS_3_-BAPT didn’t significantly change their magnetic properties. The large size of the polymers and their amorphous nature may also have no effect on the magnetic behavior of the materials.

As observed in Table 2, the saturation magnetization (Ms) values of FePS_3_-ABPT, H11, H13, and H19 are 0.430 emu/g, 0.040 emu/g, 0.044 emu/g, and 0.048 emu/g, respectively. The Ms values of nanocomposites (H11, H13, and H19) are lower than the Ms of FePS_3_-BAPT. This behavior means that the ion exchange interaction within the inter-sub-lattice of FePS_3_-BAPT and doped polymer is nearly negligible^46^. In addition, the decrease in the Ms indicates that the ability of the material to be magnetized has diminished. As it is known, the coercivity (Hc) indicates the resistance of materials to changes in magnetization. As observed in Table 2, the Hc values of the nanocomposites are lower than that of FePS_3_-BAPT, which means that the polymers inhibit the changes in the magnetization. The squareness ratio (R) value of FePS_3_-BAPT decreases after doping with PVP/PAC/SiO_2_, as presented in Table 2. This value means that a lower proportion of the saturation magnetizations are retained when the external magnetic field is removed, which is indicative of weak magnetic properties (Fig. 5), as mentioned by many authors^47,48^.

The transition from paramagnetic to diamagnetic behavior observed in the nanocomposite is attributed to combined spin-pairing and magnetic dilution effects. The doping of PVP and PAC polymers into FePS_3_-BAPT introduces nitrogen-containing functional group (-NH) and oxygen containing functional groups, carbonyl group (C = O) and hydroxyl group (-OH), that coordinate with Fe^2+^ centers, promoting electron pairing and modifying the local crystal field. In addition, the presence of diamagnetic polymers, PVP, PAC, and SiO_2_ phases reduces the density of magnetic ions and weakens magnetic exchange interactions of Fe ions through spatial separation and interfacial charge redistribution. These variations can affect the magnetic behavior of the nanocomposite by suppressing paramagnetic contributions and enhancing the overall diamagnetic response of the nanocomposite^16,49^.

**Table 2:** Magnetic parameters of FePS_3_-BAPT and nanocomposites (H11, H13, and H19).

Sample	Ms (emu/g)	Mr (emu/g)	Hc (Oe)	Rx10^−3^
FePS_3_BAPT	0.430	0.00120	28.910	346.70
SiO_2_	0.079	0.00061	35.720	129.50
H11	0.040	0.00090	22.880	44.44
H13	0.044	0.00031	26.810	141.93
H19	0.048	0.00082	15.069	58.54

Ms is the magnetization, Mr is the retentivity, Hc is the coercivity, and R is the squareness ratio.

### Optical measurement

**Fig. 6:**
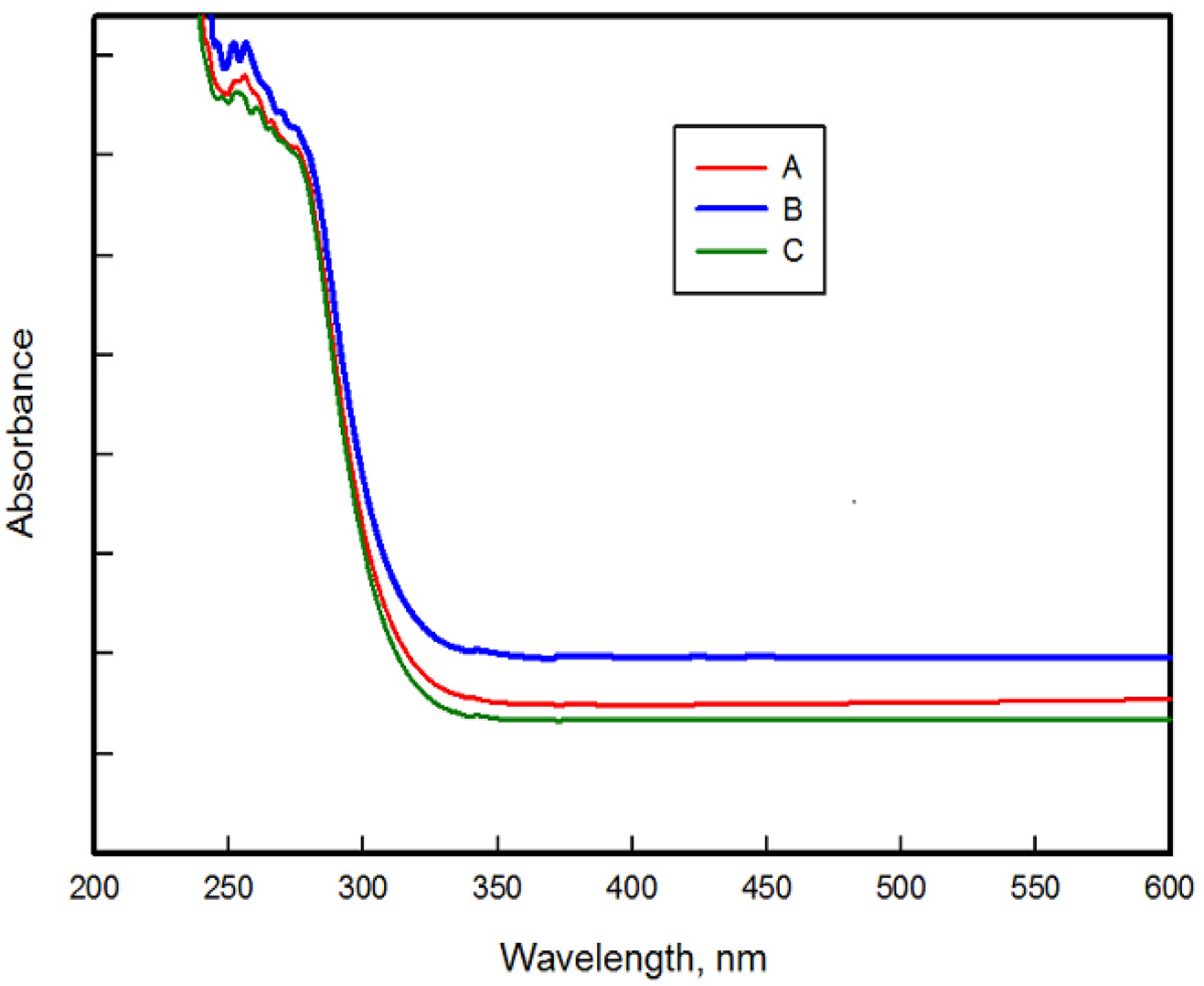
Relation between absorption spectrum as a function of wavelength of the nanocomposite.

Each material exhibits a characteristic absorption coefficient; therefore, its UV–V is absorption spectrum as a function of wavelength can be used to identify its composition, as presented in Fig. 6. The optical bad gap energies (*E*_g_) of the prepared samples H11, H13, and H19 were estimated from the cutoff wavelength and Tauc Eqs. 2^5,47,50^. This is to ensure the reproducibility of the results.

The band gap (Eg) can be estimated from the cutoff wavelength absorption edge using Eq. 3^48^.

3 $$\:{E}_{g}=\frac{hc}{\lambda\:}=\frac{1240}{\lambda\:}\:\:$$

where λ is the cutoff wavelength, h is Plank’s constant 6.26 × 10^−34^ Joule.sec, C is the light speed 3.0 × 108 m/sec.

Absorption coefficient α was calculated from absorption using Eq. 4.

4 $$\:\propto\:\:=\frac{2.303\:A}{t}\:\:\:$$

Where A is the absorbance and t is the thickness, Tauc plots were obtained from the Eq. 5^51^.

5 $$\:(\alpha\:h\upsilon\:{)}^{n\:\:}=A\left(h\upsilon\:-{E}_{g}\right)$$

**Fig. 7:**
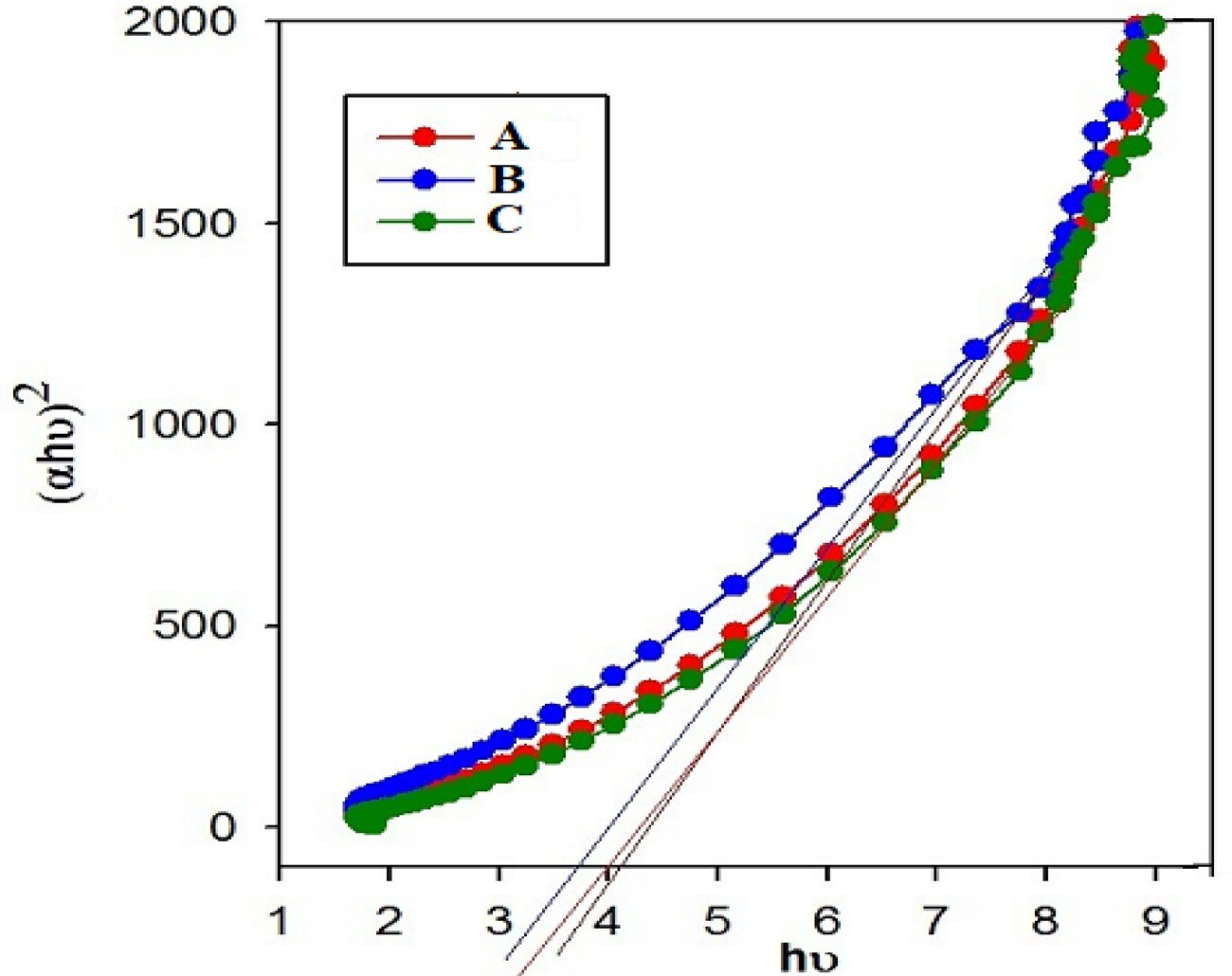
The Tauc plots for H11 (A), H13 (B), and C (H19).

Where h is the Planck’s constant, and υ is the frequency. In the Tauc equation the plot of (αhυ)^2^ versus photon energy (hυ) leads to the intersection of the linear portion with x axis give the *E*_g_, as shown in Fig. 7.

The UV–V is spectra of the three samples show a pronounced absorption peak at 255 nm, reflecting similar electronic transitions in the UV region. Their absorption edges occur at 308, 312, and 305 nm for Samples A (H11), B (H13), and C (H19), corresponding to optical band gaps of 4.03, 3.97, and 4.07 eV of both methods, respectively. The band gap has the same value from both methods. These slight variations indicate slight differences in crystallinity compared to the pristine material FePS₃-BAPT, which exhibits a band gap of 1.95 eV in a previous study^52^, the significant increase after intercalation with polyvinyl pyrrolidone (PVP), polyacrylic (PAC), and nanosilica (SiO₂) indicates a pronounced modification of the electronic structure. This change arises from the insertion of ions or molecules between the FePS_3_ layers, leading to lattice distortion, changed orbital overlap, reduced electronic delocalization, and modified charge distribution and defect states^53–60^.

The band gap widening is further attributed to strong interfacial interactions between the polymer matrix and the layered FePS₃ structure, as well as the insulating effect of the incorporated SiO₂ nanoparticles. The presence of PVP and PAC introduces polymer chains that reduce charge carrier mobility by increasing structural disorder and interrupting electronic delocalization pathways, while the SiO₂ nanoparticles act as physical barriers that restrict electron transport. Additionally, the functional groups of the BAPT modifier can coordinate with Fe centers and interact via hydrogen bonding with the polymers, further modifying the local electronic environment. As a result, the doped FePS₃-BAPT with polymers nanocomposites exhibit wide band gap behavior, highlighting their enhanced insulating behavior and potential applicability in optoelectronic, dielectric, and protective coating applications^61–63^.

## Conclusions

A new nanocomposite was prepared by doping PVP/ACA/SiO_2_ into FePS_3_-BAPT layered crystalline material. The doping process was performed using an in-situ chemical polymerization technique. The ratio of the polymers and FePS_3_-BAPT influenced the crystallinity of the resulting nanocomposite. The crystallinity percentage of the three nanocomposites (H11, H13, and H19) is about 1.5%, and the degree of amorphism of the three nanocomposites is about 98.5%. TEM measurements revealed the amorphous nature of the studied material. The doping of the PVP/ACA/SiO_2_ has affected the magnetic properties of FePS_3_-BAPT. After doping with PVP/ACA/SiO_2_, the squareness ratio value of FePS_3_-BAPT decreases. Accordingly, when the external magnetic field is removed, a lower proportion of the magnetization is retained, and the magnetic behavior has changed from paramagnetic to diamagnetic. This novel composite introduces a specific way to control the magnetization of the materials. There is an increase in the band gap energy of nanocomposites H11, H13, and H19. The strong interfacial interactions between the layered FePS₃ material and polymer matrix, and the presence of insulated SiO₂ nanoparticles have a great implication on the band gap energy. The nanocomposites of FePS_3_-BAPT/PVP/PAC/SiO_2_ is a promising multifunctional material for advanced coating applications. In addition, the combination of the polymer matrix and nanosilica, together with the modified electronic structure of the layered material FePS_3_, make it particularly suitable for corrosion protection, where it can act as both a physical shield and an active inhibitor system; this is because of combined barrier properties of the composite. The variation of the magnetic and optical properties also suggests potential application in optoelectronic and UV-blocking coatings, as well as chemical sensing platforms where surface interactions influence electronic response.

## Data Availability

Data will be made available on request.

## References

[1] Feng, X. et al. A (NH 3) x FePS 3 (A = Li, K): intercalated Fe thiophosphate via the liquid ammonia method. *Mater. Chem. Front.* **5** (6), 2715 (2021).

[2] Mayorga-Martinez, C. C. et al. Layered metal thiophosphite materials: magnetic, electrochemical, and electronic properties. *ACS Appl. Mater. Interfaces*. **9** (14), 12563 (2017). 28355055 10.1021/acsami.6b16553

[3] Chen, X. et al. A new organic-inorganic hybrid nanocomposite, BEDT-TTF intercalated into layered FePS 3. *J. Incl. Phenom. Macrocyclic Chem.* **53**, 205 (2005).

[4] Saleh, H. A., El-Shafey, S. E., Abdel-karim, A. M., Tohamy, M. S. & El-Meligi, A. A. Investigating magnetic and optical properties of layered iron phosphorus trisulfide intercalated with organic compounds. *Phys. Scr.* **100** (2), 025522 (2025).

[5] El-Meligi, A. A., Abdel-karim, A. M. & Abbas, A. A. Intercalation of layered nanomaterial with organic compounds and effect on magnetic properties. *Chem. Afr.* **7** (5), 2693 (2024).

[6] Khan, Y. (Kingston & University, 2019).

[7] Zhou, Y. & Ding, G. *Polymer Nanocomposite Materials: Applications in Integrated Electronic Devices* (Wiley, 2021).

[8] Alruwaili, A. et al. Engineered nanoparticle doping, structural, and optical innovations in polyvinyl alcohol composites for advanced optoelectronic applications. *J. Thermoplast. Compos. Mater.* **38**(11), 4012 (2025).

[9] Alziyadi, M. O., Alkabsh, A., Said, B. A. M. & Shalaby, M. S. Structural, linear/non-linear optical, and optoelectrical properties of PVB/Bi2WO6 nanocomposite for industrial applications. *J. Thermoplastic Compos. Mater.* **38**(2), 630 (2025).

[10] Alziyadi, M. O., Alkabsh, A., Said, A. M. B. & Shalaby, M. S. Effect of cadmium sulfide spheres on structural, mechanical, and optical properties of polyvinyl butyral/cadmium sulfide nanocomposite films. *J. Thermoplastic Compos. Mater.* **38**(4), 1356 (2025).

[11] Soni, G., Srivastava, S., Soni, P., Kalotra, P. & Vijay, Y. AIP Conference Proceedings, 100023. (2018).

[12] Abdah, M. A. A. M., Azman, N. H. N., Kulandaivalu, S. & Sulaiman, Y. Review of the use of transition-metal-oxide and conducting polymer-based fibres for high-performance supercapacitors. *Mater. Design*. **186**, 108199 (2020).

[13] Harb, S. V. et al. Effective corrosion protection by eco-friendly self-healing PMMA-cerium oxide coatings. *Chem. Eng. J.* **383**, 123219 (2020).

[14] Ahmed, E. H., Fathi, A. M. & El-Meligi, A. Investigating and characterizing electrical properties of polypyrrole/SiO2/FePS3 nanocomposite. *Polym. Adv. Technol.* **35**(4), e6382 (2024).

[15] Alam, M. & Chatterjee, S. Evolution of two-dimensional van der Waals materials and their applications. *J. Phys. Condens. Matter* **37**(44), 443001 (2025). 10.1088/1361-648X/ae133f 41086832

[16] Wildes, A. R. et al. Magnetic structure of the quasi-two-dimensional antiferromagnet NiPS 3. *Phys. Rev. B Condens. Matter* **92**(22), 224408 (2015).

[17] Bhadra, S., Khastgir, D., Singha, N. K. & Lee, J. H. Progress in preparation, processing and applications of polyaniline. *Prog. Polym. Sci.* **34** (8), 783 (2009).

[18] Sahoo, P. et al. In situ synthesis and properties of reduced graphene oxide/Bi nanocomposites: As an electroactive material for analysis of heavy metals. *Biosens. Bioelectron.* **43**, 293 (2013). 23334218 10.1016/j.bios.2012.12.031

[19] Lenus, S., Thakur, P., Samantaray, S. S., Narayanan, T. N. & Dai, Z. Two-dimensional iron phosphorus trisulfide as a high-capacity cathode for lithium primary battery. *Molecules* **28** (2), 537 (2023). 36677596 10.3390/molecules28020537 PMC9865732

[20] Tran, T. N., Pham, T. V. A., Le, M. L. P., Nguyen, T. P. T. & Tran, V. M. Synthesis of amorphous silica and sulfonic acid functionalized silica used as reinforced phase for polymer electrolyte membrane. *Adv. Nat. Sci. NanoSci. NanoTechnol.* **4** (4), 045007 (2013).

[21] Tantishaiyakul, V., Kaewnopparat, N. & Ingkatawornwong, S. Properties of solid dispersions of piroxicam in polyvinylpyrrolidone. *Int. J. Pharm.* **181** (2), 143 (1999). 10370210 10.1016/s0378-5173(99)00070-8

[22] Hassan, M. F. & Yusof, S. Z. M. Poly (acrylamide-co-acrylic acid)-zinc acetate polymer electrolytes: Studies based on structural and morphology and electrical spectroscopy. *Microsc. Res. Tech.* **2**(2), 30 (2014).

[23] Stefanucci, G. *Quantum Mechanics for Material Science: An Introduction* (Springer, 2024).

[24] Yang, C., Zhou, Y., Wang, X., & Zhou, Y. (2026). Sustainable High Thermal Conductivity Composites from Biomass: Bio-Based Polyimide/Microencapsulated CNTs for Green Thermal Management. ACS Applied Polymer Materials. 10.1021/acsapm.5c04260.

[25] El-Mallah, H., Abd-El Salam, M., ELesh, E. & El-Damhogi, D. Thermal annealing effect on the structural and optical characteristics of silicon phthalocyanine dichloride thin films. *Optik* **200**, 163459 (2020).

[26] Chethan, B., Prakash, H. R., Ravikiran, Y., Vijayakumari, S. & Thomas, S. Polypyyrole based core-shell structured composite based humidity sensor operable at room temperature. *Sens. Actuators B Chem.* **296**, 126639 (2019).

[27] Carter, C. B. & Williams, D. B. *Transmission Electron Microscopy: Diffraction, Imaging, and Spectrometry* (Springer, 2016).

[28] Egerton, R. F. *Electron Energy-Loss Spectroscopy in the Electron Microscope* (Springer Science & Business Media, 2011).

[29] Yang, D. & Frindt, R. Structure of polymer intercalated MnPS3 and CdPS3. *J. Mater. Res.* **15** (11), 2408 (2000).

[30] Kargar, F. et al. Phonon and thermal properties of quasi-two-dimensional FePS3 and MnPS3 antiferromagnetic semiconductors. *ACS Nano* **14**(2), 2424 (2020). 31951116 10.1021/acsnano.9b09839

[31] S’ari, M. et al. Characterization of amorphous solid dispersions and identification of low levels of crystallinity by transmission electron microscopy. *Mol. Pharm.* **18**(5), 1905 (2021). 33797925 10.1021/acs.molpharmaceut.0c00918

[32] Zidan, H. M., Abdelrazek, E. M., Abdelghany, A. M. & Tarabiah, A. E. Characterization and some physical studies of PVA/PVP filled with MWCNTs. *J. Mater. Res. Technol.* **8**(1), 904 (2019).

[33] Stuart, B. H. *Infrared Spectroscopy: Fundamentals and Applications* (Wiley, 2004).

[34] Coates, J. Interpretation of infrared spectra, a practical approach. *Encyclopedia Anal. Chem.* **12**, 10815 (2000).

[35] Robinson, I. et al. Coherent x-ray diffraction imaging of silicon oxide growth. *Phys. Rev. B* **60**(14), 9965 (1999).

[36] Salim, E., Abdelghany, A. & Tarabiah, A. E. Ameliorating and tuning the optical, dielectric, and electrical properties of hybrid conducting polymers/metal oxide nanocomposite for optoelectronic applications. *Mater. Chem. Phys.* **313**, 128788 (2024).

[37] Prasanna, S. S., Balaji, K., Pandey, S. & Rana, S. *Nanomaterials and Polymer Nanocomposites* (Elsevier, 2019).

[38] Chen, J., Wang, D. & Fu, J. Stiff yet tough, moisture-tolerant, room temperature self-healing and thermoconductive biomimetic nanocomposites. *Adv. Mater.* **37**(42), e7548. 10.1002/adma.202507548 (2025). 10.1002/adma.202507548 40736069

[39] Araujo, C. M. et al. Disorder-induced room temperature ferromagnetism in glassy chromites. *Sci. Rep.* **4**(1), 4686 (2014). 24732685 10.1038/srep04686 PMC3986702

[40] Yang, C., Zhou, Y., Wang, X. & Zhou, Y. Sustainable high thermal conductivity composites from biomass: Bio-based polyimide/microencapsulated CNTs for green thermal management. *ACS Appl. Polym. Mater.*10.1021/acsapm.5c04260 (2026).

[41] Martín-Hernández, F. & Ferré, E. C. Separation of paramagnetic and ferrimagnetic anisotropies: A review. *J. Geophys. Res. Solid Earth* **112**, B3 (2007).

[42] Kolhatkar, A. G., Jamison, A. C., Litvinov, D., Willson, R. C. & Lee, T. R. Tuning the magnetic properties of nanoparticles. *Int. J. Mol. Sci.* **14**(8), 15977 (2013). 23912237 10.3390/ijms140815977 PMC3759896

[43] Khater, G., Nabawy, B. S., Kang, J., Yue, Y. & Mahmoud, M. Magnetic and electrical properties of glass and glass-ceramics based on weathered basalt. *Silicon* **12** (12), 2921 (2020).

[44] Galluzzi, A. et al. Magnetic instabilities in the quasi-one-dimensional K2Cr3As3 material with twisted triangular tubes. *Materials* **15**(6), 2292 (2022). 35329743 10.3390/ma15062292 PMC8954554

[45] Nisticò, R., Cesano, F. & Garello, F. Magnetic materials and systems: Domain structure visualization and other characterization techniques for the application in the materials science and biomedicine. *Inorganics* **8** (1), 6 (2020).

[46] Ahmed, F., Arshi, N., Anwar, M., Danish, R. & Koo, B. H. Effect of transition metal (Co, Ni and Cu) doping on lattice volume, band gap, morphology and saturation magnetization of ZnO nanostructures. *J. Korean Phys. Soc.* **62**(10), 1479 (2013).

[47] Pang, H., Wang, H., Zhang, K., Fan, W. & Quan, W. High-precision in situ control of transverse magnetic field in ASGs based on high-frequency modulation and transient response. *IEEE/ASME Trans. Mechatron.*10.1109/TMECH.2025.3629573 (2025).

[48] Xiang, D. et al. Mechanical property enhancement of basalt fiber-reinforced epoxy composites via construction of an organic/inorganic hybrid interface. *Prog. Nat. Sci. Mater. Int.* **35**(2), 359–367. 10.1016/j.pnsc.2025.01.002 (2025).

[49] Yang, K. et al. Synthesis, structural characterization and ferrimagnetic property of MnPS_3_ intercalated with nickel (II) cyclopolyamine complex cations. *J. Solid State Chem.* **177**(11), 4300 (2004).

[50] Peng, D. et al. Hydrothermal growth of octahedral Fe_3_O_4_ crystals. *Particuology* **7**(1), 35 (2009).

[51] Yang, H. et al. Solution synthesis of layered van der Waals (vdW) ferromagnetic CrGeTe_3_ nanosheets from a non-vdW Cr_2_Te_3_ template. *J. Am. Chem. Soc.* **142**(9), 4438–4444. 10.1021/jacs.9b13492 (2020). 31976663 10.1021/jacs.9b13492

[52] Chittari, B. L. et al. Electronic and magnetic properties of single-layer MPX_3_ metal phosphorous trichalcogenides. *Phys. Rev. B* **94**(18), 184428 (2016).

[53] Abdel-karim, A. M., Fayad, A., El-Kashef, I. & Saleh, H. A. Influence of Vanadium Oxide on the optical and electrical properties of Li (oxide or fluoride) borate glasses: Influence of Vanadium Oxide on the optical and electrical properties of Li (oxide or fluoride)….. *J. Electron. Mater.* **52**(4), 2409 (2023).

[54] Sibu, G. A., Gayathri, P., Akila, T., Marnadu, R. & Balasubramani, V. Manifestation on the choice of a suitable combination of MIS for proficient Schottky diodes for optoelectronic applications: a comprehensive review. *Nano Energy*. **125**, 109534 (2024).

[55] Abbad, S. et al. Effect of silver doping on the photocatalytic activity of TiO_2_ nanopowders synthesized by the sol-gel route. *J. Environ. Chem. Eng.* **8** (3), 103718 (2020).

[56] Zhao, X., Yu, J., Cui, H. & Wang, T. Preparation of direct Z-scheme Bi_2_Sn_2_O_7_/g-C_3_N_4_ composite with enhanced photocatalytic performance. *J. Photochem. Photobiol., A* **335**, 130 (2017).

[57] Saleh, H. A., El-Shafey, S. E., Abdel-karim, A. M., Tohamy, M. S. & El-Meligi, A. A. Investigating magnetic and optical properties of layered iron phosphorus trisulfide intercalated with organic compounds. *Phys. Scr.* **100** (2), 025522 (2025).

[58] Cheng, Z. et al. High-yield production of monolayer FePS3 quantum sheets via chemical exfoliation for efficient photocatalytic hydrogen evolution. *Adv. Mater.* **30**(26), 1707433 (2018). 10.1002/adma.201707433 29782672

[59] Suwanboon, S. & Amornpitoksuk, P. Preparation of Mg-doped ZnO nanoparticles by mechanical milling and their optical properties. *Procedia Eng.* **32**, 821 (2012).

[60] Sibu, G. A., Balasubramani, V., Gayathri, P., Manthrammel, M. A. & Shkir, M. Manifesting the versatile properties of the Magnesium MOS diodes for optoelectronic applications. *Opt. Mater.* **164**, 117047 (2025).

[61] Li, K. et al. Three-stage training strategy phase unwrapping method for high speckle noises. *Opt. Express*. **32** (27), 48895–48914. 10.1364/OE.544968 (2024). 39876182 10.1364/OE.544968

[62] Xiao, C. et al. Strong and tough multilayer heterogeneous pyrocarbon based composites. *Adv. Funct. Mater.* **34**(51), 2409881. 10.1002/adfm.202409881 (2024).

[63] Hou, Z. et al. Comparative study of the micro-mechanism of functional group selection at TMD-MXene interfaces: WSe2-Ti3C2Tx heterostructures via functional group substitution from group IV, V, VI, and VII as the prototype. *Appl. Surf. Sci.* **723**, 165599. 10.1016/j.apsusc.2025.165599 (2026).

